# Association between depression and anxiety status of breast cancer patients before adjuvant chemotherapy and chemotherapy‐induced adverse events

**DOI:** 10.1002/cam4.5283

**Published:** 2022-09-26

**Authors:** Dan Lv, Bo Lan, Li Zhang, Xiaoying Sun, Min Yang, Fei Ma

**Affiliations:** ^1^ Department of Medical Oncology National Cancer Center/National Clinical Research Center for Cancer/Cancer Hospital, Chinese Academy of Medical Sciences and Peking Union Medical College Beijing China; ^2^ National Cancer Center/National Clinical Research Center for Cancer/Cancer Hospital & Shenzhen Hospital, Academy of Medical Sciences and Peking Union Medical College Shenzhen China; ^3^ Department of Medical Oncology Cancer Hospital of Huanxing Chaoyang District Beijing China; ^4^ Comprehensive Oncology Department National Cancer Center/National Clinical Research Center for Cancer/Cancer Hospital, Chinese Academy of Medical Sciences and Peking Union Medical College Beijing China

**Keywords:** adverse events, anxiety, breast cancer, chemotherapy, depression

## Abstract

**Purpose:**

Patients with breast cancer are more likely to experience psychological distress than the general population. This study aimed to explore the relationship between depression and anxiety status measured before chemotherapy and adverse events during adjuvant chemotherapy in Chinese breast cancer patients.

**Methods:**

This prospective study was conducted on 290 postoperative early‐stage breast cancer patients (response rate 96.7%) in China. Depression and anxiety status before adjuvant chemotherapy were assessed by the Hospital Anxiety and Depression Scale (HADS). Adverse events that occurred throughout the course of chemotherapy were graded and recorded according to Common Terminology Criteria for Adverse Events (CTCAE) 4.02.

**Results:**

The rates of depression and anxiety were 20.0% and 31.4%, respectively, at baseline. The incidence of grade two or higher myelosuppression induced by chemotherapy was correlated with depression before chemotherapy (*p* = 0.037). Multivariate analysis showed that the incidence of myelosuppression was significantly related to depression before chemotherapy (*p* = 0.032). There was no association between depression and anxiety status and other adverse events (*p* > 0.05).

**Conclusions:**

We observed an association between depression status in breast cancer patients receiving adjuvant chemotherapy and chemotherapy‐induced myelosuppression. Monitoring the depression status of breast cancer patients before chemotherapy may help to optimize the management of adverse events.

## Graphical abstract

We explored the relationship between depression/anxiety status and adverse events induced by adjuvant chemotherapy. Patients with depression were more likely to develop myelosuppression. Monitoring the psychological status of breast cancer patients before chemotherapy may help to optimize the management of adverse events.

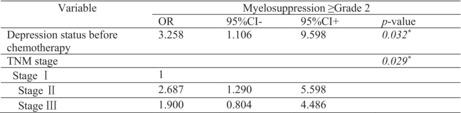

## 1 INTRODUCTION

Breast cancer is the most common malignancy among women worldwide. In 2020, more than 400,000 women were diagnosed with breast cancer in China, ranking first among new cancer cases.^1^ The treatment modalities for primary breast cancer include surgery, chemotherapy, radiotherapy and hormonal therapy, among which adjuvant chemotherapy is an important strategy to improve the survival of breast cancer patients. Although chemotherapy improves the survival rate of these patients, the aggressiveness of the chemotherapy regimen may elicit adverse side effects. In fact, cancer and treatment‐related adverse effects are major stressors among breast cancer patients undergoing treatment for the disease.^2^ Adverse events mainly included gastrointestinal events, anemia, myelosuppression, alopecia and liver‐enzymeabnormalities.

During the long treatment period, breast cancer patients often suffer from a variety of psychological distress, such as depression and anxiety.^3^ Breast cancer survivors have been found to be more likely to develop depression than the general population. Previous meta‐analyses have reported that the prevalence of anxiety and depression among breast cancer patients reached 41.9% and 32.2%, respectively.^4^, ^5^ Similarly, breast cancer survivors in China were found to experience a high level of depression and anxiety symptoms.^6^ Independent risk factors for the development of depression include age, social status, and underlying diseases.^7^, ^8^

It has long been recognized that depression and anxiety can affect the physiological function, treatment compliance,^9^ and quality of life of patients with breast cancer and may even be an important factor for survival in these patients.^10^ Depression may also cause immune dysfunction through an overactive hypothalamic–pituitary–adrenal (HPA) axis.^11^ Patients with immune dysfunction may be more likely to experience chemotherapy‐related adverse events during chemotherapy. Indeed, the relationship between depression and anxiety status and adverse events induced by chemotherapy in Chinese patients with breast cancer remains unclear. Further prospective trials exploring this relationship are essential to closing this knowledge gap.

In our study, we investigated the relationship between depression and anxiety status and adverse events during adjuvant chemotherapy in Chinese breast cancer patients with the purpose of identifying high‐risk patients through psychological state monitoring to better manage adverse events in this population.

## 2 MATERIALS AND METHODS

### 2.1 Patient selection

We identified 300 patients from a prospective, observational cohort with a primary diagnosis of breast cancer between March 1, 2019 and 30 April, 2021 in the Medical Oncology Department at the National Cancer Center in China. Ethical approval was obtained from the Institutional Review Board of the National Cancer Center/National Clinical Research Center for Cancer/Cancer Hospital. This study has been registered on ClinicalTrials.gov database.

Inclusion criteria included the following: (a) age 18–75 years, (b) had undergone surgery for breast cancer, (c) were diagnosed with stage I–III breast cancer, (d) planned to receive postoperative adjuvant chemotherapy, and (e) voluntarily provided written informed consent. Those who (1) had difficulty in understanding the questionnaire or communicating in Chinese, (2) had a history of psychiatric disorders, (3) had a history of neoadjuvant chemotherapy before surgery, or (4) had metastatic disease were excluded from the study.

#### 2.1.1 Demographic and clinical characteristics

The demographic data consisted of age, educational level, menopausal status, and family history of cancer. The clinical characteristics included tumor stage, chemotherapy regimens, and number of chemotherapy cycles.

### 2.2 Assessment instruments

The Hospital Anxiety and Depression Scale (HADS) was used to assess the psychological status of our patients, which was developed in English by Zigmond and Snaith in 1983^12^ and then translated and validated in Cantonese/Chinese in 1995 by Lam et al.^13^ Questionnaires were provided to the patients to complete before the first cycle of chemotherapy to evaluate their anxiety and depression before chemotherapy. The scale includes two parts: anxiety (anxiety, A) and depression (depression, D), with a total of 14 items—7 items with single numbers for anxiety and 7 items with double numbers for depression. Each item is scored 0–3 points. Total scores for each subscale are calculated individually, with a higher score indicating more anxiety or distress. In each of the two subscales, a score of below 8 indicates no anxiety/depression, 8–10 indicates possible anxiety/depression case, and 11–21 indicates definite anxiety/depression.^14^ Thus, based on previous research, we adopted a threshold subscale score of 8 for anxiety and depression in this study.

All patients received 4 or 6–8 cycles of conventional chemotherapy regimens include anthracycline‐, taxane‐, or combined anthracycline‐ and taxane‐based chemotherapy specified by the National Comprehensive Cancer Network (NCCN) guidelines (Version 3.2018 to Version 1.2021), based on their disease states, and for Her2‐positive patients, anti‐Her2 has been added. Dexamethasone and 5‐hydroxytryptamine receptor (5HT3) prophylactic antiemetic therapy were administered before each cycle of chemotherapy for moderate and low emetogenic chemotherapy. Dexamethasone, 5HT3, and neurokinin‐1 (NK‐1) receptor antagonist prophylactic antiemetic therapy were administered to patients receiving hyperemetic chemotherapy. Adverse events (gastrointestinal events, anemia, myelosuppression, alopecia, elevation of transaminase) in each chemotherapy cycle were continuously monitored and graded according to the Common Terminology Criteria for Adverse Events (CTCAE) version 4.0.2. We recorded and analyzed the most serious grades of each adverse event in patients. Finally, the most serious adverse event during chemotherapy was recorded for follow‐up analysis.

### 2.3 Statistical analysis

Statistical analysis was performed using SPSS 22.0 software. Cronbach's α values were calculated to assess the internal consistency of each questionnaire. Chi‐squared analysis was used to examine whether there was a significant relationship between adverse events induced by chemotherapy and psychological symptoms and demographic and clinical characteristics. We used a logistic regression model to investigate potential risk factors for adverse events. A probability value of *p* < 0.05 was considered statistically significant.

## 3 RESULTS

### 3.1 Patient characteristics

A total of 300 breast cancer patients fulfilled the inclusion criteria, and all patients provided informed consent. Eight patients dropped out due to discontinuation of treatment at our hospital, and two patients were excluded due to incomplete questionnaires. Thus, a total of 290 valid cases were analyzed, and information on the demographic and clinical characteristics of these patients were presented in Table 1. The Eastern Cooperative Oncology Group (ECOG) performance scores of all patients were 0–1. Internal consistency measurements of the HADS subscale questionnaires before chemotherapy yielded Cronbach's alpha coefficients of 0.883, respectively. Overall, the mean age of the patients was 47.09 ± 9.45 years, and 40.7% were older than 50 years. And 5.9% of patients received anthracycline‐based chemotherapy, 40.3% of patients received taxane‐based chemotherapy and 53.8% of patients received combined anthracycline‐ and taxane‐based chemotherapy. Among them, more than half of the patients were diagnosed with stage II cancer (50.7%), and 85.5% of patients received 6–8 cycles of chemotherapy decided by their physicians.

**TABLE 1:** Demographics and clinical characteristics of the patients (*N* = 290)

Characteristic	Number of patients
Age (years)	
<50	172 (59.3%)
≥50	118 (40.7%)
Received anthracycline chemotherapy	
Yes	173 (59.7%)
No	117 (40.3%)
TNM stage	
Stage I	76 (26.2%)
Stage II	148 (51.0%)
Stage III	66 (22.8%)
Number of chemotherapy cycles	
4	42 (14.5%)
6–8	248 (85.5%)

### 3.2 Prevalence of anxiety and depression

The rates of depression and anxiety were 20.0% (58/290) and 31.4% (91/290) at baseline. The mean (95% confidence interval [CI]) HADS depression score was 4.63 (1.06, 8.20), and the mean anxiety score was 6.00 (2.41, 9.59).

### 3.3 Correlation between anxiety or depression and adverse events

First, the most common adverse events observed in our study were grade 2 or higher myelosuppression (84.1%). A total of 26.9% of the patients experienced grade 2 or higher gastrointestinal events, while more than half of the patients (54.5%) experienced grade 1 or higher elevated transaminase. The incidences of grade 2 or higher anemia and alopecia were 22.1% and 16.2%, respectively. Afterward, we analyzed the differences of common adverse events induced by chemotherapy (including gastrointestinal events, anemia, alopecia, and elevation of transaminase) in different psychological status before chemotherapy, and we found that the occurrence of grade two or higher myelosuppression during chemotherapy in patients with depression was higher than that in patients without depression before chemotherapy (93.1% vs. 81.9%, *p* = 0.037). No statistically significant result was found between anxiety and myelosuppression during chemotherapy (*p* = 0.234). There was no statistical significance between depression and anxiety status and other adverse events, including gastrointestinal events, anemia, alopecia, and elevation of transaminase (*p* > 0.05) (Table 2).

**TABLE 2:** Univariate analysis of common adverse events induced by chemotherapy and psychological symptoms

Characteristic	Depression status before chemotherapy		χ^2^	*p*‐value	Anxiety status before chemotherapy		χ^2^	*p*‐value
	Yes	No			Yes	No		
Myelosuppression ≥Grade 2								
Yes	54 (22.1%)	190 (77.9%)	4.367	0.037*	80 (32.8%)	164 (67.2%)	1.415	0.234
No	4 (8.7%)	42 (91.3%)			11 (23.9%)	35 (76.1%)		
Gastrointestinal events ≥Grade 2								
Yes	21 (26.9%)	57 (73.1%)	3.196	0.074	27 (34.6%)	51 (65.4%)	0.519	0.471
No	37 (17.5%)	175 (82.5%)			64 (30.2%)	148 (69.8%)		
Anemia ≥Grade 2								
Yes	16 (25.0%)	48 (75.0%)	1.283	0.257	17 (26.6%)	47 (73.4%)	0.885	0.347
No	42 (18.6%)	184 (81.4%)			74 (32.7%)	152 (62.3%)		
Alopecia ≥Grade 2								
Yes	12 (25.5%)	35 (74.5%)	1.073	0.300	19 (40.4%)	28 (59.6%)	2.132	0.144
No	46 (18.9%)	197 (81.1%)			72 (29.6%)	171 (70.4%)		
ALT/AST ≥Grade 1								
Yes	32 (20.3%)	126 (79.7%)	0.014	0.906	50 (31.6%)	108 (68.4%)	0.011	0.915
No	26 (19.7%)	106 (80.3%)			41 (31.1%)	91 (68.9%)		

*Note*: *p‐*values were obtained from chi‐squared tests for categorical variables.

Abbreviations: ALT, alanine aminotransferase; AST aspartate aminotransferase.

* *p* < 0.05.

In the multivariate analysis, after adjusting for age, prechemotherapy depression status, chemotherapy regimens, 8th edition of AJCC Tumor‐Node‐Metastasis (TNM) stage, and number of chemotherapy cycles (univariate analysis between myelosuppression and demographic and clinical factors were shown in Table Table S1), we found that prechemotherapy depression status was independently associated with the incidence of grade two or higher myelosuppression induced by chemotherapy, and this was statistically significant (*p* = 0.032). In other words, these patient populations are more likely to develop myelosuppression (odds ratio [OR] = 3.258 [1.106–9.598]). The results of the multivariate analysis of the correlation between depression status before chemotherapy and myelosuppression induced by chemotherapy are shown in Table 3.

**TABLE 3:** Multivariate analysis for the correlation of pre‐chemotherapy depression status and myelosuppression induced by chemotherapy

Variable	Myelosuppression ≥Grade 2			
	OR	95% CI−	95% CI+	*p*‐value
Depression status before chemotherapy	3.258	1.106	9.598	0.032*
TNM stage				0.029*
Stage I	1			
Stage II	2.687	1.290	5.598	
Stage III	1.900	0.804	4.486	

*Note*: ORs and 95% CIs were from polytomous logistic regression models.

Abbreviations: CI, confidence interval; OR, odds ratio.

* *p* < 0.05.

## 4 DISCUSSION

We conducted this study to explore the relation between psychological distress and adverse events induced by chemotherapy. We used the HADS scale to assess psychological status as it has been used as a criterion for evaluating depression and anxiety in previous studies.^15^, ^16^, ^17^, ^18^ In this study, we analyzed 290 patients with breast cancer in China, and the rate of depression and anxiety were 20.0% and 31.4%, which were comparable to previous reports.^3^, ^19^, ^20^ The results reported in other studies also indicate the importance of assessing the mental health of such patients throughout the entire cancer treatment process.^21^, ^22^ Post hoc analysis showed that depression before chemotherapy was associated with the incidence of myelosuppression during the entire chemotherapy process. This indicates that patients with depression in these populations are more likely to develop myelosuppression during the course of chemotherapy. Thus, it is very important to screen the psychological status of breast cancer patients before chemotherapy. We found no correlation between anxiety and adverse events during chemotherapy and there was no in‐depth mechanism exploration at present. Further mechanism research may be carried out in the future.

In this study, the patients received conventional chemotherapy regimens, and no granulocyte colony‐stimulating factor was used for primary prevention of myelosuppression. A total of 84.1% of patients had grade 2 or higher myelosuppression, which is similar to previous study results.^23^ The occurrence of myelosuppression in our study reflected the true condition of the patients after receiving chemotherapy. Therefore, the conclusions drawn by our study have high credibility and may offer guidance regarding clinical decision‐making.

The possible reason for these conclusions may be that psychological distress in the form of depression and anxiety can cause immune dysfunction. In previous studies, psychological distress is considered to dysregulate immune function in several ways, including the exacerbation of chronic inflammation, the suppression of protective immunity, and the enhancement of immunosuppressive mechanisms.^24^, ^25^, ^26^, ^27^ Activation of the HPA axis, which is well recognized in individuals with depression, inhibits immune system activity by releasing cortisol and disrupts the immune microenvironment.^28^, ^29^ Therefore, breast cancer patients experiencing depression are more prone to myelosuppression during chemotherapy. In addition, depression, anxiety, or a combination of both is associated with increased recurrence and mortality in patients with breast cancer.^10^ Hence, oncologists should try to identify patients with depression before chemotherapy and take measures when necessary to relieve psychological distress.

Some retrospective studies have been conducted to explore the role of psychological distress in chemotherapy‐induced nausea and vomiting, and the results suggest that anxiety plays a role in the perception, frequency, intensity, and severity of nausea and other chemotherapy‐induced side effects.^30^ In our study, univariate analysis showed that depression and anxiety were not associated with gastrointestinal events. This result is different from the results of previous studies.^31^, ^32^ The reason may be because in the present study, the patients received preventive antiemetic treatment before chemotherapy, which reduced the incidence of gastrointestinal adverse events to a nonsignificant level. This also suggests that preventive antiemetic treatment can relieve nausea and vomiting in patients. In addition, there was no statistically significant association between psychological status and elevated transaminases, anemia, and alopecia. This indicates that depression and anxiety in breast cancer patients do not affect the incidence of such adverse events.

In this work, we demonstrated, for the first time, that depression status is an independent factor associated with myelosuppression induced by chemotherapy in Chinese breast cancer patients. This work measured the depression and anxiety status of breast cancer patients before the course of chemotherapy. Patients with depression before chemotherapy were found to be more likely to develop grade two or higher myelosuppression during chemotherapy. These findings indicate that oncologists should make efforts to measure the depression status of breast cancer patients before chemotherapy. Nevertheless, proper psychological care is still necessary after screening.^33^ Before starting chemotherapy, it is necessary for oncologists to consider not only the patient's tumor development and disease experience but also their psychological status, including depression and other psychological symptoms.^34^, ^35^ Moreover, oncologists and nurses should perform intervention measures when patients with severe psychological symptoms are identified to ensure that they can successfully complete chemotherapy, which is of great significance for preventing tumor recurrence and improving patient prognosis.

As intended, this study assessed the psychological status of breast cancer patients being monitored at the National Cancer Center/National Clinical Research Center for Cancer/Cancer Hospital and identified factors associated with chemotherapy‐induced myelosuppression, which will help oncologists and nurses offer better support to breast cancer patients. Nevertheless, some limitations should be noted. First, this study was conducted at a single institute. Second, the population size was relatively small. Additional studies with larger populations should be conducted to improve the validity of our conclusions in the future. Third, our study was merely an observational study, without intervention on patients, thus it was impossible to draw any conclusions about causality.

In conclusion, we demonstrated in this exploratory study that the depression status of breast cancer patients was correlated with the incidence of grade two or higher myelosuppression throughout the course of chemotherapy, and populations with prechemotherapy depression were more likely to develop myelosuppression. These findings suggest that oncologists should focus more attention on monitoring the depression status of breast cancer patients before chemotherapy, which might help to improve the management of adverse events, and ensure the safety of patients.

## AUTHOR CONTRIBUTIONS

**Dan Lv:** Conceptualization (lead); data curation (lead); formal analysis (lead); methodology (lead); resources (lead); software (lead); supervision (equal); validation (lead); visualization (equal); writing – original draft (lead); writing – review and editing (equal). **Bo Lan:** Conceptualization (equal); data curation (equal); formal analysis (equal); funding acquisition (equal); resources (equal); supervision (equal); validation (equal); visualization (equal); writing – original draft (equal); writing – review and editing (equal). **Li Zhang:** Data curation (equal); formal analysis (equal); funding acquisition (supporting); methodology (equal); resources (equal); validation (equal); visualization (equal); writing – original draft (equal); writing – review and editing (equal). **Xiaoying Sun:** Data curation (equal); formal analysis (equal); methodology (equal); validation (equal); visualization (equal); writing – original draft (equal); writing – review and editing (equal). **Min Yang:** Data curation (equal); formal analysis (equal); methodology (equal); validation (equal); visualization (equal); writing – original draft (equal); writing – review and editing (equal). **Fei Ma:** Conceptualization (lead); data curation (equal); formal analysis (equal); methodology (equal); project administration (lead); supervision (lead); validation (equal); visualization (equal); writing – original draft (equal); writing – review and editing (lead).

## CONFLICT OF INTEREST

The authors declare that they have no conflicts of interest.

## ETHICS APPROVAL

The study was approved by the Institutional Review Board of the National Cancer Center/National Clinical Research Center for Cancer/Cancer Hospital.

## INFORMED CONSENT

All participants provided informed consent.

## CLINICAL TRIAL REGISTRATION NUMBER

The study is registered (ClinicalTrials.gov identifier: NCT05055375).

## Supporting information


Table S1

## Data Availability

The datasets used and/or analyzed during the current study are available from the corresponding author upon reasonable request.

## References

[1] Sung H , Ferlay J , Siegel RL , et al. Global cancer statistics 2020: GLOBOCAN estimates of incidence and mortality worldwide for 36 cancers in 185 countries. CA Cancer J Clin. 2021;71(3):209‐249. 33538338 10.3322/caac.21660

[2] Jim HS , Andrykowski MA , Munster PN , Jacobsen PB . Physical symptoms/side effects during breast cancer treatment predict posttreatment distress. Ann Behav Med. 2007;34(2):200‐208. 17927558 10.1007/BF02872674

[3] So WK , Marsh G , Ling WM , et al. Anxiety, depression and quality of life among Chinese breast cancer patients during adjuvant therapy. Eur J Oncol Nurs. 2010;14(1):17‐22. 19734087 10.1016/j.ejon.2009.07.005

[4] Hashemi SM , Rafiemanesh H , Aghamohammadi T , et al. Prevalence of anxiety among breast cancer patients: a systematic review and meta‐analysis. Breast Cancer. 2020;27(2):166‐178. 31828585 10.1007/s12282-019-01031-9

[5] Pilevarzadeh M , Amirshahi M , Afsargharehbagh R , Rafiemanesh H , Hashemi SM , Balouchi A . Global prevalence of depression among breast cancer patients: a systematic review and meta‐analysis. Breast Cancer Res Treat. 2019;176(3):519‐533. 31087199 10.1007/s10549-019-05271-3

[6] Lam WW , Bonanno GA , Mancini AD , et al. Trajectories of psychological distress among Chinese women diagnosed with breast cancer. Psychooncology. 2010;19(10):1044‐1051. 20014074 10.1002/pon.1658

[7] Wang F , Liu J , Liu L , et al. The status and correlates of depression and anxiety among breast‐cancer survivors in eastern China: a population‐based, cross‐sectional case‐control study. BMC Public Health. 2014;14:326. 24708474 10.1186/1471-2458-14-326 PMC3997219

[8] Yang H , Brand JS , Fang F , et al. Time‐dependent risk of depression, anxiety, and stress‐related disorders in patients with invasive and in situ breast cancer. Int J Cancer. 2017;140(4):841‐852. 27859142 10.1002/ijc.30514

[9] DiMatteo MR , Lepper HS , Croghan TW . Depression is a risk factor for noncompliance with medical treatment: meta‐analysis of the effects of anxiety and depression on patient adherence. Arch Intern Med. 2000;160(14):2101‐2107. 10904452 10.1001/archinte.160.14.2101

[10] Wang X , Wang N , Zhong L , et al. Prognostic value of depression and anxiety on breast cancer recurrence and mortality: a systematic review and meta‐analysis of 282,203 patients. Mol Psychiatry. 2020;25(12):3186‐3197. 32820237 10.1038/s41380-020-00865-6 PMC7714689

[11] Reiche EM , Morimoto HK , Nunes SM . Stress and depression‐induced immune dysfunction: implications for the development and progression of cancer. Int Rev Psychiatry. 2005;17(6):515‐527. 16401550 10.1080/02646830500382102

[12] Zigmond AS , Snaith RP . The hospital anxiety and depression scale. Acta Psychiatr Scand. 1983;67(6):361‐370. 6880820 10.1111/j.1600-0447.1983.tb09716.x

[13] Lam CL , Pan PC , Chan AW , Chan SY , Munro C . Can the hospital anxiety and depression (HAD) scale be used on Chinese elderly in general practice? Fam Pract. 1995;12(2):149‐154. 7589936 10.1093/fampra/12.2.149

[14] Arving C , Glimelius B , Brandberg Y . Four weeks of daily assessments of anxiety, depression and activity compared to a point assessment with the hospital anxiety and depression scale. Qual Life Res. 2008;17(1):95‐104. 18026852 10.1007/s11136-007-9275-4

[15] Snaith RP , Taylor CM . Rating scales for depression and anxiety: a current perspective. Br J Clin Pharmacol. 1985;19(Suppl 1):17s‐20s. 3994903 10.1111/j.1365-2125.1985.tb02737.x PMC1463499

[16] Friedman S , Samuelian JC , Lancrenon S , Even C , Chiarelli P . Three‐dimensional structure of the hospital anxiety and depression scale in a large French primary care population suffering from major depression. Psychiatry Res. 2001;104(3):247‐257. 11728614 10.1016/s0165-1781(01)00309-2

[17] Herrmann C . International experiences with the hospital anxiety and depression scale—a review of validation data and clinical results. J Psychosom Res. 1997;42(1):17‐41. 9055211 10.1016/s0022-3999(96)00216-4

[18] Annunziata MA , Muzzatti B , Bidoli E , et al. Hospital anxiety and depression scale (HADS) accuracy in cancer patients. Support Care Cancer. 2020;28(8):3921‐3926. 31858249 10.1007/s00520-019-05244-8

[19] Civilotti C , Acquadro Maran D , Santagata F , Varetto A , Stanizzo MR . The use of the distress thermometer and the hospital anxiety and depression scale for screening of anxiety and depression in Italian women newly diagnosed with breast cancer. Support Care Cancer. 2020;28(10):4997‐5004. 32036468 10.1007/s00520-020-05343-x

[20] Ozalp E , Soygür H , Cankurtaran E , Turhan L , Akbiyik D , Geyik P . Psychiatric morbidity and its screening in Turkish women with breast cancer: a comparison between the HADS and SCID tests. Psychooncology. 2008;17(7):668‐675. 17992701 10.1002/pon.1286

[21] Hopwood P , Haviland J , Mills J , Sumo G , Bliss JM . The impact of age and clinical factors on quality of life in early breast cancer: an analysis of 2208 women recruited to the UK START trial (standardisation of breast radiotherapy trial). Breast. 2007;16(3):241‐251. 17236771 10.1016/j.breast.2006.11.003

[22] Takahashi T , Hondo M , Nishimura K , et al. Evaluation of quality of life and psychological response in cancer patients treated with radiotherapy. Radiat Med. 2008;26(7):396‐401. 18769996 10.1007/s11604-008-0248-5

[23] Tian H , Qin W , Wu W , et al. Effects of traditional Chinese medicine on chemotherapy‐induced myelosuppression and febrile neutropenia in breast cancer patients. Evid Based Complement Alternat Med. 2015;2015:736197. 26347793 10.1155/2015/736197 PMC4549530

[24] Antoni MH , Dhabhar FS . The impact of psychosocial stress and stress management on immune responses in patients with cancer. Cancer. 2019;125(9):1417‐1431. 30768779 10.1002/cncr.31943 PMC6467795

[25] Blume J , Douglas SD , Evans DL . Immune suppression and immune activation in depression. Brain Behav Immun. 2011;25(2):221‐229. 20955778 10.1016/j.bbi.2010.10.008 PMC3025086

[26] Dhabhar FS . The short‐term stress response—mother nature's mechanism for enhancing protection and performance under conditions of threat, challenge, and opportunity. Front Neuroendocrinol. 2018;49:175‐192. 29596867 10.1016/j.yfrne.2018.03.004 PMC5964013

[27] Zhang L , Pan J , Chen W , Jiang J , Huang J . Chronic stress‐induced immune dysregulation in cancer: implications for initiation, progression, metastasis, and treatment. Am J Cancer Res. 2020;10(5):1294‐1307. 32509380 PMC7269780

[28] Borgi M , Collacchi B , Ortona E , Cirulli F . Stress and coping in women with breast cancer:unravelling the mechanisms to improve resilience. Neurosci Biobehav Rev. 2020;119:406‐421. 33086128 10.1016/j.neubiorev.2020.10.011

[29] Keller J , Gomez R , Williams G , et al. HPA axis in major depression: cortisol, clinical symptomatology and genetic variation predict cognition. Mol Psychiatry. 2017;22(4):527‐536. 27528460 10.1038/mp.2016.120 PMC5313380

[30] Grassi L , Berardi MA , Ruffilli F , et al. Role of psychosocial variables on chemotherapy‐induced nausea and vomiting and health‐related quality of life among cancer patients: a European study. Psychother Psychosom. 2015;84(6):339‐347. 26402426 10.1159/000431256

[31] Kamen C , Tejani MA , Chandwani K , et al. Anticipatory nausea and vomiting due to chemotherapy. Eur J Pharmacol. 2014;722:172‐179. 24157982 10.1016/j.ejphar.2013.09.071 PMC3880638

[32] Molassiotis A , Yam BM , Yung H , Chan FY , Mok TS . Pretreatment factors predicting the development of postchemotherapy nausea and vomiting in Chinese breast cancer patients. Support Care Cancer. 2002;10(2):139‐145. 11862503 10.1007/s00520-001-0321-4

[33] Mitchell AJ , Vahabzadeh A , Magruder K . Screening for distress and depression in cancer settings: 10 lessons from 40 years of primary‐care research. Psychooncology. 2011;20(6):572‐584. 21442689 10.1002/pon.1943

[34] Antoni MH , Wimberly SR , Lechner SC , et al. Reduction of cancer‐specific thought intrusions and anxiety symptoms with a stress management intervention among women undergoing treatment for breast cancer. Am J Psychiatry. 2006;163(10):1791‐1797. 17012691 10.1176/ajp.2006.163.10.1791 PMC5756627

[35] Scheier MF , Helgeson VS , Schulz R , et al. Interventions to enhance physical and psychological functioning among younger women who are ending nonhormonal adjuvant treatment for early‐stage breast cancer. J Clin Oncol. 2005;23(19):4298‐4311. 15994143 10.1200/JCO.2005.05.362

