# Supplementary material for: Association between depression and anxiety status of breast cancer patients before adjuvant chemotherapy and chemotherapy‐induced adverse events
Source: Cancer Med. 2022 Sep 26;12(4):4794–800. doi: 10.1002/cam4.5283 (PMC9972093; doi:10.1002/cam4.5283)
Supplement: Supplementary file 1 — Table S1 [file CAM4-12-4794-s001.docx]

Table S1. Univariate analysis for myelosuppression > or = Grade 2 and demographic and clinical factors.

| Characteristic | myelosuppression > or = Grade 2 | Myelosuppression< Grade 2 | *n* | *p*-value |
| --- | --- | --- | --- | --- |
| Age(years) |  |  |  |  |
| <50 | 140(81.4%) | 32(18.6%) | 172 | 0.123 |
| ≥50 | 104(88.1%) | 14(11.9%) | 118 |  |
| TNM stage |  |  |  |  |
| Stage Ⅰ | 58(75.3%) | 19(24.7%) | 77 | *0.035^*^* |
| Stage Ⅱ | 130(88.4%) | 17(11.6%) | 147 |  |
| Stage Ⅲ | 56(84.8%) | 10(15.2%) | 66 |  |
| Number of chemotherapy cycles received |  |  |  |  |
| 4 | 37(88.1%) | 5(11.9%) | 42 | 0.448 |
| 6-8 | 207(83.5%) | 41(16.5%) | 248 |  |
| Chemotherapy regimens |  |  |  |  |
| Anthracycline-based chemotherapy | 16(94.1%) | 1(5.9%) | 17 |  |
| Taxane--based chemotherapy | 95(81.2%) | 22(18.8%) | 117 | 0.337 |
| Combined anthracycline and taxane-based chemotherapy | 133(85.3%) | 23(14.7%) | 156 |  |

**p* < 0.05.
